# Accuracy of uploadable pedometers in laboratory, overground, and free-living conditions in young and older adults

**DOI:** 10.1186/1479-5868-9-143

**Published:** 2012-12-11

**Authors:** Christopher J Dondzila, Ann M Swartz, Nora E Miller, Elizabeth K Lenz, Scott J Strath

**Affiliations:** 1 Physical Activity and Health Research Laboratory, University of Wisconsin-Milwaukee, P.O. Box 413, Milwaukee, WI, 53201, USA; 2 The College at Brockport, State University of New York, 350 New Campus Dr., Brockport, NY, 14420, USA

**Keywords:** Walking, Step-counter, Precision, Accuracy, Motion sensor

## Abstract

**Purpose:**

The purpose of this study was to examine the accuracy of uploadable pedometers to accurately count steps during treadmill (TM) and overground (OG) walking, and during a 24 hour monitoring period (24 hr) under free living conditions in young and older adults.

**Methods:**

One hundred and two participants (*n*=53 aged 20–49 yrs; *n*=49 aged 50–80 yrs) completed a TM protocol (53.6, 67.0, 80.4, 93.8, and 107.2 m/min, five minutes for each speed) and an OG walking protocol (self-determined “< normal”, “normal”, and “> normal” walking speeds) while wearing two waist-mounted uploadable pedometers (Omron HJ-720ITC [OM] and Kenz Lifecorder EX [LC]). Actual steps were manually tallied by a researcher. During the 24 hr period, participants wore a New Lifestyles-1000 (NL) pedometer (standard of care) attached to a belt at waist level over the midline of the left thigh, in addition to the LC on the belt over the midline of the right thigh. The following day, the same procedure was conducted, replacing the LC with the OM. One-sample *t*-tests were performed to compare measured and manually tallied steps during the TM and OG protocols, and between steps quantified by the NL with that of the OM and LC during the 24 hr period. Mean error step scores (MES, criterion – device) and 95% Limits of Agreement (LoA) were calculated.

**Results:**

There were no significant differences between the OM and tallied steps for any of the TM speeds for either the young or older adult groups. The LC significantly underestimated steps for the young adult group during the 53.6 m/min TM speed (MES 31.4 [14.5, 48.3]) and during the OG < normal walking speed (MES 12.0 [0.9, 23.1] (*p*<0.01 for both age groups). The LC also significantly underestimated steps for the older adult group during the TM speeds of 53.6 m/min (MES 64.5 [45.6, 83.4]), 67.0 m/min (MES 15.1 [6.1, 24.0]), and 80.4 m/min (MES 3.2 [0.6, 5.9]) (*p*<0.01 for all speeds), in addition to the OG < normal walking speed (MES 14.7 [−13.3, 42.6] (*p*<0.01). The OM reported significantly lower steps during the 24 hr period for the young adult group by 949.1 steps (t=6.111, *p*<0.025) and for the older adult group by 612.9 steps (t=2.397, *p*<0.025).

**Conclusion:**

Both the OM and LC pedometers were more accurate as TM and OG walking speed increased. The OM significantly underestimated steps during the 24 hr compared with a standard of care evaluation. Overall, both uploadable pedometers appear acceptable to use in young or old age groups to measure walking behavior.

## Background

Regular physical activity has long been shown to be host to a variety of benefits related to chronic conditions and diseases, such as diabetes, obesity, hypertension, and heart disease across a variety of populations [1]. One commonly employed method in physical activity promotion is the utilization of pedometers, an inexpensive device that objectively monitors ambulatory physical activity. Numerous studies have documented beneficial health outcomes through pedometer-based physical activity interventions [2-4].

There are various styles of pedometers with differing technology to quantify steps, such as spring-levered and piezoelectric sensors. As the technology of these devices has advanced, newer pedometer models are able to connect to a computerized interface, such as a desktop computer, allowing ambulatory physical activity behavior to be uploaded. For instance, the Omron and New Lifestyle pedometer brands have such capabilities. Uploadable pedometers aim to further expand on the potential to increase and maintain activity habits to users by offering additional information and features. Such features could include individualized feedback and progress updates on daily walking behaviors, setting visual walking targets and how users compare to such targets, offering behavioral feedback cues based upon uploaded walking behaviors, all of which can be insightful during interventional purposes [5].

Numerous pedometer brands have been tested for their ability to quantify walking behavior, with the majority of such research focusing on younger, healthy adults [6-8]. Research examining the accuracy of such devices in the older adult population is more sparse [9-11], and collectively a paucity of data across all population ages exists evaluating newer technology uploadable pedometers. Therefore, the primary purpose of this study was to test the accuracy of two uploadable pedometers in measuring walking behavior during laboratory, overground, and free living activity in a group of community dwelling young and older adults.

## Methods

### Participants

A convenience sample of 102 adults participated in this study across two age groups: 20 – 49 years (*n*=53) and 50 – 80 years (*n*=49). All participants were recruited through word of mouth, posted fliers, and media announcements. Inclusion criteria consisted of being in general good health and able to participate in regular physical activity, and aged 20 – 80 years. Exclusionary criteria consisted of the inability to safely walk and/or run on a treadmill or around an indoor track, and/or the use of a required walking aid. One hundred and eleven individuals enrolled in the study, with 9 individuals dropping out. Of the 9 that dropped out (3 in the 20–49 years group and 6 in the 50–80 years group) the reason was an overall lack of time to finish all study visits. There were no demographic differences between those that finished the study and those that dropped out (data not shown). All participants were informed of potential risks and benefits of participation and signed an informed consent document approved by the University Institutional Review Board prior to study enrollment.

### Study design

Participation in this study consisted of four separate visits. Visit one consisted of study explanation, obtaining informed consent, the completion of general demographics, and a treadmill walking protocol while wearing assigned pedometers for evaluation. During visit two participants were asked to complete an over-ground (track) variable speed walking protocol while wearing assigned pedometers. All participants were then asked to return for a third visit, and during this visit participants were instructed on how to wear the monitors for a 24 hour (24 hr) monitoring period. Finally, participants came back to return all pedometers following the 24 hr monitoring period, for a fourth and final visit. Each visit was separated by a minimum of 24 hours. All participants completed study visits in order.

### Study measures

Participants had their body height (to the nearest 0.1 cm) and body mass (to the nearest 0.01 kg) measured with no shoes and minimal clothing via a calibrated physician’s scale and stadiometer (Detecto, Kansas City, MO). Body mass index (BMI) was calculated by dividing body mass (kg) by height squared (m^2^). Waist circumference was measured in duplicate and averaged to the nearest 0.1 cm at the narrowest area of the trunk between the iliac crest and inferior rib using a tension-sensitive tape measure. Individual stride length was determined by a standardized process. Participants began with feet together and walked to a marker (103.6 m away). The distance was measured from the marker to the heel of the first foot that crossed the marker and added to the 103.6 m walked. Total distance walked was divided by the total number of steps taken to determine stride length. The accuaracy of steps quantified by the uploadable pedometers was examined across treadmill walking, over ground walking, and free living conditions.

### Uploadable pedometers

#### Omron HJ-720ITC pedometer

The Omron HJ-720ITC pedometer (OM; Omron Corporation, Kyoto, Japan) is a waist-worn pedometer that uses a piezoelectric sensor to quantify steps and distance, including the ability to extrapolate caloric expenditure. The device is capable of storing up to 7 days of data for immediate retrieval in its display, and up to 41 days of data in its memory, which can be obtained by uploading the information to a computer. Information is automatically stored and reset at midnight each day.

#### Kenz lifecorder EX pedometer

The Kenz Lifecorder pedometer (LC; Suzuken Co. Ltd., Nagoya, Japan) is a waist-worn pedometer that uses a piezoelectric sensor to quantify steps and calories, as well as demarcate recorded steps between light, moderate, and vigorous intensities. The device is able store 7 days of information for immediate retrieval in its display, and up to 200 days of information stored in internal memory, which can be accessed by uploading the pedometer to a computer.

### Treadmill walking protocol

Participants walked on a treadmill (TrackMaster TMX22, Newton, KS) at fixed speeds of 53.6, 67.0, 80.4, 93.8, and 107.2 m/min for 5-minutes at each protocol speed, or up until a point that participants reached 85% of estimated maximal heart rate. Each treadmill speed was verified with a digital tachometer (Shimpo Instruments, Itasca, IL) and found to be within ± 0.1%.

Prior to engaging in the treadmill walking protocol, each participant was fitted with the Kenz Lifecorder EX pedometer on the midline of the right thigh and the Omron HJ-720ITC pedometer on the midline of the left thigh, both secured to a belt at the level of the waist. Both anatomical site locations are supported and suggested by the manufacturers. During the treadmill walking protocol actual steps were tallied by a researcher using a hand-tally counter. In between walking protocol speeds participants straddled the treadmill, so that pedometer steps could be recorded from both the LC and the OM. This permitted a pre and post walking step count to be recorded from the LC and OM, to yield steps accumulated during each protocol speed.

### Overground walking protocol

On a separate day participants returned to complete a track variable speed walking protocol. Each participant walked once around an indoor track (394 m) at three different self-determined speeds. These speeds were required to be < normal, normal, and > normal walking speeds. Hence, these speeds were variable across participants and self-selected. During this time each participant wore the LC and OM pedometers in midline of the right and left thigh, respectively, affixed to a belt at the level of the waist. During each track walking speed, total distance was recorded to calculate speed, and actual steps were manually tallied by a researcher. For all participants tested, walking speed increased across the three variable conditions of < normal, normal, and > normal. These speed allocations were designed to have each individual walk across their own self-determined walking speed range that may be typical of overground walking for them in a naturalistic lifestyle setting.

## 24 Hour monitoring period

A random subset of 20 participants agreed to engage in a 24 hr evaluation of each uploadable pedometer. Participants were instructed to wear pedometers for 24 hours, except when sleeping, and when in contact with water (such as bathing, showering, or swimming). For one 24 hr observation period participants wore the LC on the right midline of the thigh (same position as the laboratory testing) and on the left midline of the thigh participants placed a New Lifestyles NL-1000 (NL) pedometer (New Lifestyles, Inc., Warminster, PA), both secured on a belt at waist level. Although no gold-standard device exists to measure accrued steps on a daily basis under free-living conditions, the NL series pedometer is an industry standard pedometer, heavily utilized for interventional purposes. The NL series pedometer has an extensive empirical backing in the literature for validity and reliability, able to accurately quantify increasing walking intensity activities (<2% error) [12], while retaining high intramodel reliability (0.99) [13]. Accordingly, NL pedometers have been shown to be substantially more sensitive to walking behavior during observational periods, compared to other pedometers [10]. As such, the NL was worn as a standard of care comparison, not a criterion comparison, with the LC. Each participant was given instructions to write down day starting steps on the pedometer, and day ending steps on each pedometer. The difference constituted daily steps from each brand, the LC and the NL. The following day, this was repeated, with the OM pedometer worn on the left midline of the thigh, (same position as the laboratory testing) and on the right midline of the thigh participants wore the NL pedometer, both secured by a belt at waist level. Schneider et al. [12] and others [6] have shown high correlation coefficients (r=0.99) for NL brand pedometers when worn on the left and right side of the body, thus justifying the use of the NL-1000 pedometer to be worn on both the left and right side across days.

### Statistical analysis

All statistical analyses were performed utilizing SPSS 19.0 for Windows (Chicago, IL). Descriptive statistics are expressed as mean ± standard deviation. For each treadmill walking and overground walking activity an error score was computed for each participant, by subtracting the estimate (pedometer) from the criterion (manually tallied) and compared with zero. Error scores of zero would indicate that there was no difference between the pedometer and criterion measure. Positive error scores represent underestimates, and negative error scores represent overestimates. The MES scores for each treadmill walking and overground walking speed for both uploadable pedometers were tested using one-sample *t-*tests and Bonferroni corrections for multiple comparisons. Limits of agreement (LoA) are presented as +/− 1.96 Standard Deviations from the MES. For the 24-hour observation the NL-1000 served as the standard of care comparison against the LC and OM. MES and 95% LoA were again calculated and statistically tested using one-sample *t*-tests.

## Results

### Participant characteristics

Participant demographics are shown in Table 1. Those participants in the 50–80 year old group were significantly older than those in the 20–49 year old group (by study design). Participants’ mean BMI values were classified as overweight [14]. However, the mean values for waist circumference in both age categories are classified as low risk for disease development [14]. Participants in the 20–49 year category were marginally taller, and had a slightly longer stride length compared to the older age group. The decline in stride length from the young age category to the older age category is consistent with previous research [15].

**Table 1:** Physical and descriptive characteristics of the participants (Mean±SD)

**Variable**	**20-49 yrs (*n*=53)**	**50-80 yrs (*n*=49)**
Age (yrs)	32.9±10.8	65.4±6.9*
Height (cm)	169.9±12.2	167.1±8.7
Mass (kg)	77.4±23.0	72.8±14.7
WC (cm)	81.1±12.4	85.8±12.2
BMI (kg·m^-2^)	25.6±5.1	25.9±4.0
Stride length (cm·step^-1^)	72.6±7.1	69.9±9.9

*Note.* WC, waist circumference; BMI, body mass index; * significantly different than young group (*p*<0.01).

### Pedometer accuracy: treadmill walking

**Figure 1:**
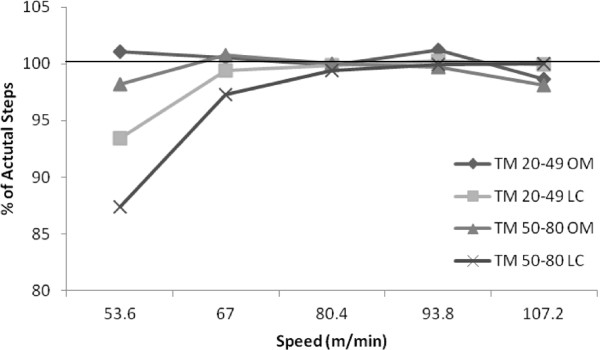
**Percent accuracy for pedometers across treadmill walking speeds.** The OM and LC pedometers were worn during treadmill speeds of 53.6, 67.0, 80.4, 93.8, and 107.2 m/min, for five minutes at each speed. A researcher manually counted the steps the participant engaged in, and the pedometers’ accuracy in measuring the actual steps (percent of total steps measured) were calculated by the following equation: (actual steps – measured pedometer steps) * 100.

Mean error scores and LoA for both age groups during the treadmill walking are reported in Table 2. In general, both pedometers became more accurate in measuring steps as the treadmill walking speed increased. The OM pedometer was most accurate at the 80.4 m/min stage, whereas the LC pedometer was most accurate at the 107.2 m/min stage. The OM pedometer was least accurate at the 107.2 m/min stage, although the MES of 8.4 (1.3%) and 12.4 (1.9%) steps (younger adult category and older adult category, respectively) were not significant. The LC pedometer was least accurate at the 53.6 m/min stage, with this pedometer significantly underestimating accumulated steps by 31 (6.6%) and 65 (12.6%) for the younger adult and older adult category, respectively. Furthermore, the LC pedometer significantly underestimated accumulated steps in the older adult category at both 67.0 m/min and 80.4 m/min by 15.1 (2.7%) and 3.2 (0.6%) steps, respectively (see Figure 1).

**Table 2:** Mean error step scores and limits of agreement during treadmill walking protocol

**Pedometer/Group**	**53.6 m/min**	**67.0 m/min**	**80.4 m/min**	**93.8 m/min**	**107.2 m/min**
OM: 20–49 yrs	−5.4 (−13.4, 2.5)	−2.7 (−6.4, 1.0)	0.8 (−2.9, 4.4)	−6.6 (−17.7, 4.5)	8.4 (0.2, 16.6)
OM: 50–80 yrs	9.7 (−4.9, 24.3)	−4.5 (−18.2, 9.1)	−0.2 (−2.2, 1.8)	1.9 (−0.9, 4.7)	12.4 (−1.3, 26.2)
LC: 20–49 yrs	31.4* (14.5, 48.3)	3.2 (−0.3, 6.7)	0.5 (−0.5, 1.6)	−1.2 (−5.4, 3.0)	0.3 (−0.6, 1.1)
LC: 50–80 yrs	64.5* (45.6, 83.4)	15.1* (6.1, 24.0)	3.2* (0.6, 5.9)	0.3 (−2.6, 3.1)	−0.2 (−1.5, 1.1)

*Note.* OM = Omron HJ-720ITC, LC= Kenz Lifecorder EX. Negative scores represent an overestimation of steps whereas positive scores represent an underestimation of steps; * significantly different than zero *p*<0.01.

### Pedometer accuracy: overground walking

**Figure 2:**
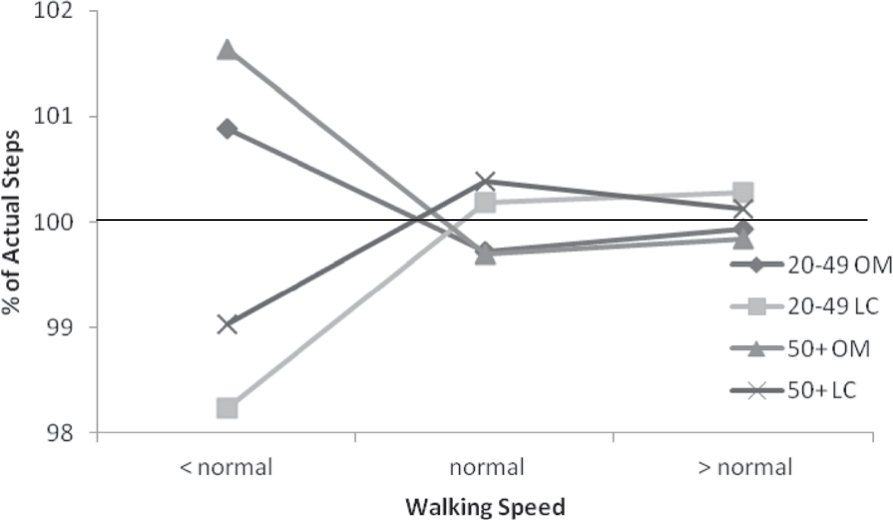
**Percent accuracy for pedometers across overground walking speeds.** The OM and LC pedometers were worn during three self selected speeds (“< normal”, “normal”, and “> normal”) on an indoor track over the distance of 394m. A researcher manually counted the steps the participant engaged in, and the pedometers’ accuracy in measuring the actual steps (percent of total steps measured) were calculated by the following equation: (actual steps – measured pedometer steps) * 100.

Mean error scores and LoA for both age groups during the overground walking are reported in Table 3. Collectively, the MES for both the OM and LC pedometers (across both age groups) decreased as the walking speed increased from < normal to normal, and from normal to > normal walking speeds. Both pedometers exhibited large MES across both age groups during the < normal walking speed. The largest, and only significant, MES was shown by the LC pedometer in the younger adult category during the < normal walking speed, underestimating steps by 12.0 (1.8%) (see Figure 2).

**Table 3:** Mean error step scores and limits of agreement during overground walking protocol

**Pedometer/Group**	**<Normal walking speed**	**Normal walking speed**	**>Normal walking speed**
OM: 20–49 yrs	−4.7(−11.0, 1.7)	1.7(−6.2, 9.5)	0.5(−4.7, 5.7)
OM: 50–80 yrs	0.3(−31.8, 32.5)	1.9(−8.0, 11.9)	0.9(−2.9, 4.6)
LC: 20–49 yrs	12.0*(0.9, 23.1)	−0.9(−3.0, 1.1)	−1.3(−2.9, 0.3)
LC: 50–80 yrs	14.7*(−13.3, 42.6)	−1.1(−11.1, 8.9)	−0.7(−2.1, 0.7)

*Note.* OM = Omron HJ-720ITC, LC= Kenz Lifecorder EX;* significantly different than zero *p*<0.01.

### Pedometer accuracy and precision: 24 hr observation

During the 24 hr free-living periods, the younger and older groups had mean step measurements of 9470.2 and 9074.7 for the OM pedometer, and 10649.5 and 11094.8 for the LC pedometer during the 24 hr monitoring period. The MES scores and LoA for both age groups during the 24 hr observation period is presented in Table 4. The OM pedometer significantly underestimated steps for the younger and older adult age groups by 949.1 (13.0%) and 612.9 (6.8%) steps, respectively.

**Table 4:** Mean error step scores and limits of agreement during 24 hour observation period

**Pedometer**	**20-49 yrs (*n*=10)**	**50-80 yrs (*n*=10)**
OM	949.1* (597.8, 1300.4)	612.9* (34.4, 1191.4)
LC	−305.1 (−709.9, 99.7)	−38.0 (−936.6, 860.6)

*Note.* OM = Omron HJ-720ITC, LC= Kenz Lifecorder EX;* significantly different than zero *p*<0.025.

## Discussion

There is a plethora of research that demonstrates pedometers’ ability to promote increases in ambulatory activity. Efforts have been made to make pedometers more user friendly, such as reducing the user’s need to frequently record daily steps. Uploadable pedometers aim to achieve such capabilities, and also have the potential to provide the user with continual and individualized feedback. There is limited research on the accuracy of such pedometers, and this lack of knowledge resonates more so in the older adult population. The results of the current study showed that the OM and LC were increasingly accurate in their ability to quantify steps in both participant age groups as both treadmill walking speed and overground walking speed increased. The LC significantly underestimated steps during the 53.6 m/min stage for the younger adult group, and during the 53.6, 67.0, and 80.4 m/min stages for the older adult group. The LC also significantly underestimated steps for the younger adult group during the less than normal walking speed in the overground walking trial. During the 24 hr observation period, the OM pedometer significantly underestimated steps for both young and older adult groups.

Similar to previously published research, the OM pedometer generally became increasingly more accurate as walking speed increased on the treadmill. Using the same treadmill speeds as the current study, the Omron HJ-105 has been shown to have the largest percent error in measuring steps at 2.0 mph, with increasing accuracy as treadmill speed increased [16]. A study by Foster et al. showed the Omron HF-100 to be accurate (>98%) in quantifying steps at speeds greater than 2.0 mph [17]. Likewise, studies examining the validity of the Omron HJ-122, Omron HJ-720ITC, and Omron HJ-113 have demonstrated the pedometers’ high accuracy for quantifying steps at increasing speeds above 2.0 mph [18,19]. These results, collectively with those of the current study, indicate that Omron pedometers become increasingly accurate in assessing ambulatory activity above slow walking speeds. One study, however, indicates otherwise. Crouter and colleagues showed the Omron HJ-105 to significantly overestimate steps at treadmill walking speeds at 4.0 mph (the fastest speed of the protocol) [6]. The trend for increasing accuracy of the Kenz Lifecorder during increasing treadmill speeds resonates that of previous research, which shows the slowest walking speeds to have the largest mean error [6,20]. Current study results also show significant underestimations at the 2.0 mph stage, but were much more evident in older adults. Although two additional walking speeds significantly underestimated steps in older adults in the current study, the corresponding percent errors were similar to previously reported results in younger adults [20]. Overall, both the Omron HJ-720ITC and Kenz Lifecorder examined in the present study represent suitable options for walking behaviors assessed via treadmill, with minor decrements in accuracy at slower walking speeds.

The results of the current study for overground trials provides further credence that the pedometers examined in the current study are generally accurate in their measurements of walking activity. The OM of the current study reported more accurate step measurements than that of reported by Schneider et al., who reported an underestimation of 19.0 steps over a distance nearly identical to that of the current study [12]. Even at the slowest walking cadence of the present protocol, the largest mean error was an overestimation of 4.7 steps (for the young adult group). These results are similar with those presented by Holbrook et al. [21]. They assessed the validity of two Omron brand pedometers over 100 m walking trials, reporting absolute percent errors less than 2% for the HJ-151 for their slow, moderate, fast, and self-selected speed trials, and less than or equal to 2% for the HJ-720ITC for the same trials. The OM in the current study had percent errors less than 2% for the < normal walking speed, and less than 1% for the normal and > normal walking speeds. Currently, there exists limited research on the validity of the Kenz Lifecorder during overground walking. We are aware of one study that assessed such during self-selected walking paces on a track, which showed a less than 1% error in steps [12], which are very similar to the normal walking speed results of the current study. Overall, the OM and LC pedometers exhibited increasing accuracy during faster walking cadences during the OG protocol, a trend similar to that of the TM protocol.

The OM pedometer recorded less steps compared with the NL standard of care comparison in both the young age group and the older age group during the 24 hr observation period. Silcott et al. also examined the Omron HJ-720ITC pedometer during day long observation periods in a sample of adults aged similar (mean 31.3-46.2 years) to that of the young adult category in the present study (20–49 years), and showed the pedometer to significantly underestimate steps [22]. Collectively, their results are likely due to the hardware of the Omron pedometer, as they only quantify steps after movement of four seconds or more. The magnitude of difference in error was approximately 13% for the young age group and 7% for the older age group in the current study. Although such differences are marginal, it highlights that a degree of caution is needed when comparing daily values of accrued steps across different pedometer brands.

There are several limitations of the current study that warrant mention. The populations used were all generally healthy, so it may not be appropriate to extend the results previously stated to diseased, those with gait impairments, or obese populations. Due to feasibility, the NL was used as the comparison variable for comparing OM and LC measured steps during the observation period rather than employing a manually tallied count. The current study does, however, fill an important void in the literature, by examining the validity of uploadable pedometers in both young and old age groups across laboratory, overground, and free-living activities.

## Conclusions

Both the OM and LC pedometers were more accurate as TM and OG walking speed increased. The OM significantly underestimated steps during the 24hr monitoring period compared with a standard of care evaluation, and highlights that caution is needed when comparing total accrued step per day values across different pedometer brands. Overall, both uploadable pedometers appear acceptable to use in young or old age groups to measure walking behavior.

## Abbreviations

OM: Omron HJ-720ITC pedometer; LC: Kenz Lifecorder EX pedometer; TM: Treadmill walking; OG: Overground walking.

## Competing interests

The authors declare that they have no competing interests.

## Authors’ contributions

SJS and AMS conceptualized the study. NEM and EKL collected the data. CJD performed data analysis and drafted the manuscript. All authors read and approved the final manuscript.
